# Key Performance Analysis of Emulsified Asphalt Cold Recycling Mixtures of the Middle Layer of Pavement Structure

**DOI:** 10.3390/ma16041613

**Published:** 2023-02-15

**Authors:** Jun Li, Mingliang Li, Hao Wu

**Affiliations:** Research Center of Road, Research Institute of Highway Ministry of Transport, Beijing 100088, China

**Keywords:** road engineering, cold in-place recycling, emulsified asphalt, high temperature stability, cracking resistance, moisture susceptibility

## Abstract

In the maintenance engineering of asphalt pavement, it is often encountered that both the surface and middle layers are damaged and need to be maintained. The cold in-place recycling technology can be used to simultaneously treat multi-layer diseases and reduce the waste of pavement materials. The cold in-place recycling mixture is rarely used for high layer of pavement structure in high-grade highway. In the supporting practical engineering, the emulsified asphalt cold in-place recycling mixtures were paved as the middle layer of pavement structure by the laying of an overlay. In order to comprehensively evaluate the material performances, coring samples were drilled after cold recycling pavement opening to traffic, and different performance tests were carried out based on the coring samples. The newly paved SMA mixtures were set as the control group. The high temperature stability of cold recycling mixture was analyzed by dynamic creep test and MMLS3 accelerated loading test. Then, the cracking resistance of cold recycling mixture was studied by semi-circular bending test. Finally, the effect of curing time on splitting strength of cold recycling mixture was measured, and the moisture susceptibility was analyzed by dry–wet splitting test and freeze–thaw splitting test. The test results showed that the high temperature stability of cold recycling mixture was worse than SMA mixture. For the cold recycling mixture, the deformation value at the early stage and deformation rate at the stable stage were larger than SMA mixture in the accelerated loading process, and shear failure at high temperature occurred earlier. The cracking resistance of cold recycling mixture was worse than SMA mixture because of the aging effect of the old asphalt and adverse influence of the added cement binder. The effect of curing time on splitting strength of cold recycling mixture was significant, and two stable periods of early strength were, respectively, reached after curing 3 days and 7 days. The indexes of moisture susceptibility, including dry–wet splitting strength ratio and freeze–thaw splitting strength ratio, were obviously lower than that of SMA mixture, and the test values not up to the standard requirement existed. For the emulsified asphalt cold in-place recycling mixture, the improvement of material performances should be focused on, especially the moisture susceptibility. In the research, the emulsified asphalt cold in-place recycling mixtures were acceptably used as the middle layer of maintenance pavement structure. The reliable discussions were summarized based on coring samples collected from real-life road sections. The case can provide guidance and reference for similar engineering applications.

## 1. Introduction

Asphalt pavement durability is affected by many factors, including vehicle loading and climate, during the process of transportation, which makes its pavement performance decay continuously until it needs to be repaired because of pavement diseases. The performance of the upper layer or the middle layer of some pavement sections is seriously attenuated. As a result, a large number of reclaimed asphalt pavement (RAP) materials will be produced to cause waste because of the use of conventional milling and resurfacing methods for maintenance, which does not conform to the maintenance concept of green and environment-friendly protection. For pavements that need multi-layer maintenance, it is economical and effective to repair pavement diseases and improve the overall structural performance by using the cold in-place recycling technology to carry out multi-layer synchronous recycling and subsequent construction of a new overlay, however, in which the cold recycling layer is used as the middle layer of the maintenance pavement structure [1,2]. In comparison with the conventional low-layer cold recycling, the high-layer cold recycling mixture takes more vehicle loading and poses higher requirements for comprehensive pavement performance of cold recycling mixtures [3,4].

Many factors influence the pavement performance of cold recycling mixtures. Therefore, reasonable design of material composition characteristics, such as raw material, volume characteristics, and key control parameters, can give full play to the performance of cold recycling mixtures [5,6,7,8,9]. RAP material is the biggest contributor of cold recycling mixture as its properties, including field moisture content, asphalt binder condition, content, source, aggregate gradation, etc., are closely related to the performances the of cold recycling mixture [10,11,12,13]. In the field of soil or recycled demolition wastes stabilization for subbase and base, asphalt emulsion is widely used, and the adhesive force of the mixture is enhanced after the demulsification [14,15]. Asphalt emulsion is one of the commonly used binder materials for cold recycling mixtures. The polymer type, ionic charge, and demulsification rate of emulsified asphalt all have significant influence on the performance of cold recycling mixture [16]. Different types of asphalt emulsion have corresponding applicability, so it is necessary to determine the optimal emulsion type according to the application scenarios. The cementitious stabilization agent is added into cold recycling mixtures for the objective of increasing the bearing strength and the compressive strength [17]. Cement is the most commonly used among all of the cementitious stabilization agents, but the moderate amount of the agent should be determined based on a proper mix design to achieve the best in-service performance [18]. The influences of curing conditions on the consolidation behavior of cold recycling mixtures were analyzed, in the laboratory and on site, respectively [19,20]. With the idea that the curing temperature has an important influence on the consolidation behavior of the cold recycling mixture, the measure was proposed to accelerate its consolidation by heating [21]. The improvement of pavement performance also stands out as one of the key directions in the research of cold recycling mixtures. The pavement loading test or triaxial compression test was used to test the rutting resistance of cold recycling mixtures with different amounts of cementing materials, leading to the conclusion that the rutting resistance is the best when the amount of cementing materials ranges between 2% and 2.5% [22,23]. The cold recycling mixtures used for pavement should also have good durability to avoid pavement diseases that will shorten the service life of the pavement. The cracking resistance and fatigue resistance of the asphalt mixture can be effectively characterized by fracture energy and flexibility index calculated by the semi-circular bending test [24,25,26,27,28]. The indirect tensile fatigue test was used to study the fatigue resistance of cold recycling mixtures [29]. The moisture susceptibility of cold recycling mixtures under immersion or freeze–thaw condition is also very important, which can be improved by optimizing the gradation and adding the appropriate amount of emulsified asphalt, cement, and fibers [30,31,32,33,34].

The cold in-place recycling mixture is mainly used for low-grade highway surface layers or high-grade highway base layers, and relevant research has been studied by scholars at home and abroad. The cold in-place recycling mixture is rarely applied to high-grade highway high-layer surface layers because of the limitations of the material performances and construction quality of on site. In addition, the research subjects of relevant studies are mostly cold recycling mixtures prepared in the laboratory, with few scholars conducting systematic research on the service performance of cold recycling pavements in service in practical engineering. The durability is directly affected by the rutting resistance, cracking resistance, and moisture susceptibility of the cold recycling mixtures in the duration of their service. In this paper, the application effects of cold recycling mixtures in the middle layer of pavement structure are comprehensively evaluated. The research subjects consisted of two sections of emulsified asphalt cold in-place recycling pavement. By drilling core samples on the pavement, various pavement performances of the cold recycling mixture were analyzed through different test methods.

## 2. Test Design

### 2.1. Cold in-Place Recycling Maintenance Scheme

**Figure 1:**
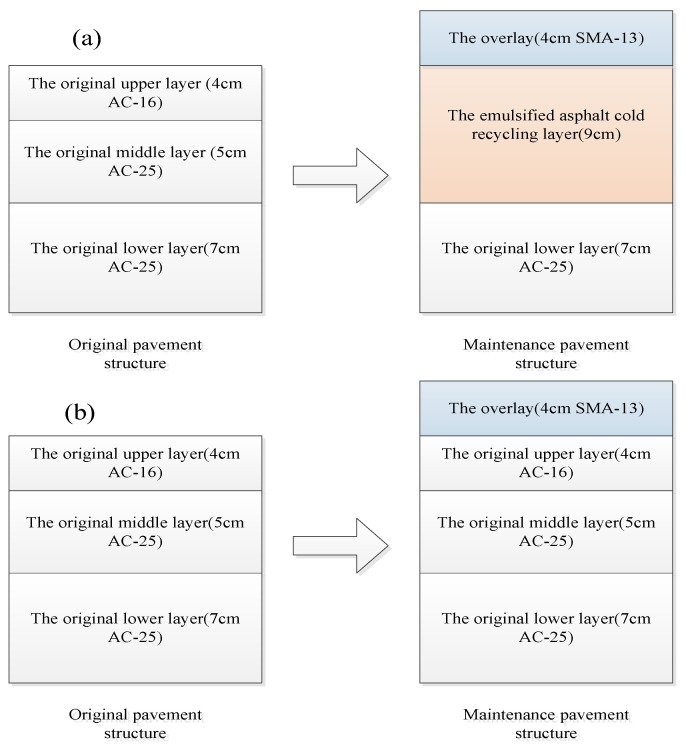

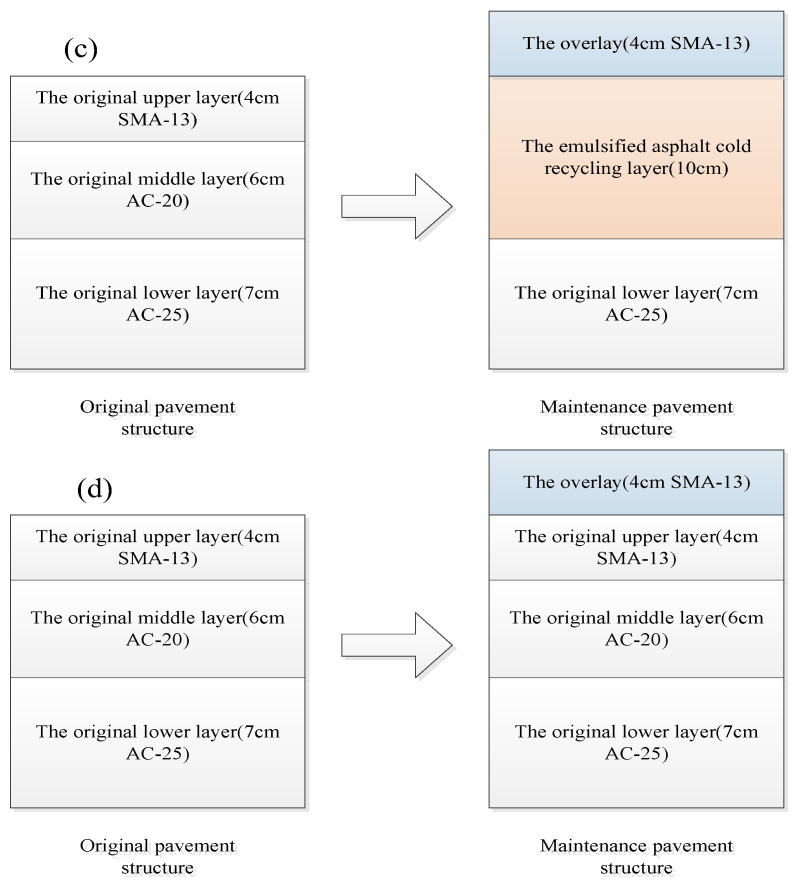
Different maintenance schemes of asphalt pavement. (**a**) Maintenance scheme of traffic lane (Section 1); (**b**) maintenance scheme of emergency lane (Section 1); (**c**) maintenance scheme of traffic lane (Section 2); (**d**) maintenance scheme of emergency lane (Section 2).

Two sections of emulsified asphalt cold in-place recycling pavement were selected in expressway practical engineering. The construction was completed in July 2019 with the pavement core drilling conducted in December 2019, followed by related tests. The maintenance scheme, that milling of 1 cm on the upper layer of the original pavement and cold recycling of the upper and middle layers synchronously followed by the laying of an overlay, was designed in the traffic lane, based on which the cold recycling layer was used as the middle layer of the maintenance pavement structure. For the cold recycling mixture, besides adding 3.5% of SBR emulsified asphalt (by mass of RAP materials, the same below), 2.0% of cement and 2.79% of water (ensuring the optimum moisture content) were added into the cold recycling mixture. These additives can improve the workability and enhance the comprehensive performances of the cold recycling mixture. The emergency lane was directly paved with an overlay due to its original good performance. The maintenance schemes are shown in Figure 1. In the figures, SMA stands for stone matrix asphalt, and AC stands for asphalt concrete. The numbers of 13, 16, 20, and 25 represent nominal maximum aggregate size of 13.2 mm, 16 mm, 19 mm, and 26.5 mm, commonly used in China.

### 2.2. Test Methods

#### 2.2.1. High-Temperature Stability Test

(1) Dynamic creep test

A dynamic creep test was conducted based on asphalt mixture performance tester in accordance with the standard of AASHTO T 378-17 [35]. The test temperature was set at 60 °C initially and then adjusted to 55 °C due to the rapid destruction of the core samples. The load was applied at 0.7 MPa, with a half-sine wave as the loading waveform and a loading cycle of 1 s (consisting of a half-sine pressure load for 0.1 s and an interval for 0.9 s).

The dynamic creep test curve was composed of three phases: migration, stabilization, and damage. The model formulas for each stage were given in Equations (1)–(3). In the migration phase, the accumulation of permanent deformation was rapid, but the accumulation rate decreased slowly. In the stabilization phase, the accumulation rate of permanent deformation remained generally constant, while the deformation accumulation was slow. As for the damage phase, the deformation accumulation began to grow fast with a sharply rising growth rate. The number of repeated load actions for the third phase was determined as the flow number (FN), representing the inflection point at which the permanent deformation of the asphalt mixture entered a rapid-developing phase. In this paper, the FN was adopted as a dynamic creep test indicator for evaluating the ultimate high-temperature stability of asphalt mixtures.

(1) $$\varepsilon_{p}{=a\times N}^{b}$$

(2) $$\varepsilon_{p}{=\varepsilon }_{\mathrm{ps}}{+c\times (N-N}_{ps})$$

(3) $$\varepsilon_{p}{=\varepsilon }_{\mathrm{st}}{+d\times (e}^{{f(N-N}_{st})}-1)$$

where *ε*_p_ donates the accumulative permanent strain, *ε*_ps_ represents the permanent strain at the beginning of the second phase, *ε*_st_ stands for the permanent strain at the beginning of the third phase, *N* means the number of load actions, *N*_ps_ corresponds to the number of load actions at the beginning of the second phase. *N*_st_ is the number of load actions at the beginning of the third phase, and the letters *a*, *b*, *c*, *d*, *e*, and *f* are material constants related to the test conditions.

(2) MMLS3 accelerated loading test

The accelerated loading test was carried out based on 1/3 model mobile load simulator (MMLS3). Simulated loading was performed with a maximum load of 2.7 KN, equivalent to a 0.7 MPa load. The test was conducted at a maximum loading speed of 9 km/h (7200 times/h) and in a water bath heating environment of 60 °C. More details of the test setup are available in the relevant literature [36]. The rutting depth of the samples at different numbers of loading was recorded to reflect the high temperature and anti-deformation of the asphalt mixture. The indicators of the MMLS3 accelerated loading test included the deformation after 100,000 times of loading (*RD*_10_) and the deformation ratio between 100,000 and 200,000 times of loading (*DS*_20−10_), with the former characterizing the high-temperature stability of the asphalt mixture at the early stage of the loading while the latter characterizing the long-term high-temperature stability of the asphalt mixture. The calculation for *DS*_20−10_ is shown in Equation (4).

(4) $${\mathrm{DS}}_{20-10}=\frac{{\mathrm{RD}}_{20}{-\mathrm{RD}}_{10}}{20-10}\times 100$$

where *DS*_20−10_ is the deformation rate between 100,000 and 200,000 times of loading (10^−3^ μm/time), *RD*_10_ donates the deformation after 100,000 times of loading (mm), and *RD*_20_ means the deformation after 200,000 times of loading (mm).

#### 2.2.2. Anti-Cracking Performance Test

**Figure 2:**
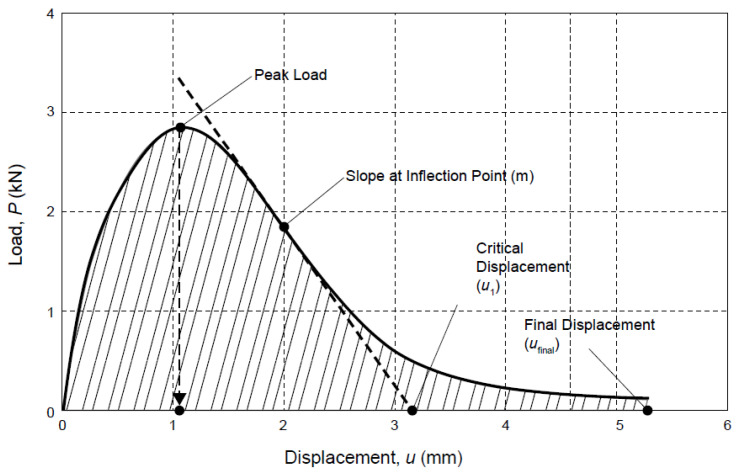
Typical test loading curve of SCB test [37].

A semi-circular bending (SCB) test was conducted at the temperature of 15 °C and a loading rate of 50 mm/min in accordance with the standard of AASHTO TP 124-16, with the typical test loading curve shown in Figure 2 (from TP 124-16, AASHTO Provisional Standards, published by the American Association of State Highway and Transportation Officials, Washington, DC, USA, used with permission) [37]. In Figure 2, the letter *u*_1_ represents the intersection of the post-peak slope with the displacement-axis. A straight line is drawn connecting the inflection point and displacement axis with a slope *m*. The letter *u*_final_ means displacement at the 0.1 kN cut-off load. The intersection of the arrow with the displacement-axis stands for the displacement at peak load. The test indicators consisted of fracture energy and flexibility index (FI). The damage form of the specimen in the SCB test is similar to the cracking process of asphalt pavement. The SCB test can be used to predict the crack propagation law of asphalt pavement. The fracture energy represents energy required to create a unit surface area of a crack. Therefore, the calculated fracture energy indicates an asphalt mixture’s overall capacity to resist cracking-related damage. Generally, a mixture with higher fracture energy can resist greater stresses with higher damage resistance. A greater value of both indicators implied better anti-cracking performance of the asphalt mixture. Fracture energy *G_f_* reflected the total energy absorbed from the material from the state of intactness to fracture, which was calculated by the ratio of the fracture power to the toughness zone area in Equation (5).

(5) $$G_{f}=\frac{W_{f}}{{\mathrm{Area}}_{\mathrm{lig}}}$$

where *G_f_* represents the fracture energy (J/m^2^). *Area_lig_* means the toughness zone area (m^2^), which was calculated as shown in Equation (6):

(6) $${\mathrm{Area}}_{\mathrm{lig}}=\left(r-a\right)\times t$$

where the letter *r* is the radius of the sample (m). The letter *a* means the crack length (m). The letter *t* stands for sample thickness (m).

*W_f_* means the work of fracture (J), which can be calculated using the integral equation below.

(7) $$W_{f}=\int^{\;}\mathrm{Pdu}$$

where the letter *P* means the applied load (N). The letter *u* is the average displacement of the load (m). The letter *du* represents differentiation of the displacement *u*.

The flexibility index was calculated as follows:

(8) $$\mathrm{FI}=\frac{G_{f}}{\left|m\right|}\times A$$

where FI indicates the flexibility index (dimensionless). *G_f_* means the fracture energy (J/m^2^). |m| is the absolute value of the inflection point slope of the load displacement curve after the peak (kN/mm). The letter *A* denotes the unit conversion coefficient, which is 0.01.

#### 2.2.3. Moisture Susceptibility Test

The moisture susceptibility of emulsified asphalt cold recycling mixtures was evaluated via the wet–dry splitting test and freeze–thaw splitting test. The former adopted the wet–dry splitting strength ratio as the evaluation indicator, which is the percentage of the splitting strength in water immersion for 24 h to that in normal conditions (Equation (9)). The splitting test under normal conditions was carried out based on the T0716 in accordance with Chinese specification of JTG E20-2011 [38]. In the 24 h water immersion splitting test, the samples were completely immersed in a constant temperature water bath at 25 °C for 22 h in advance, after which the splitting strength was tested according to the requirements of the splitting test under normal conditions. The wet–dry splitting strength ratio of emulsified asphalt cold recycling mixtures should not be less than 80% for heavy-load and above transportation purposes.

(9) $$TSR_{1}=\frac{{\bar{R}}_{\mathrm{wet}}}{{\bar{R}}_{\mathrm{dry}}}\times 100$$

where *TSR_1_* means the dry–wet splitting strength ratio (%). ${\bar{R}}_{\mathrm{wet}}$ represents the average value of splitting tensile strength of effective samples after immersion curing (MPa). ${\bar{R}}_{\mathrm{dry}}$ denotes the average value of splitting tensile strength of effective samples after normal curing (MPa).

As the evaluation indicator for the freeze–thaw splitting test, the freeze–thaw splitting strength ratio was calculated in Equation (10) and performed based on the T0729. The freeze–thaw splitting strength ratio of emulsified asphalt cold recycling mixtures should not be less than 75% for heavy-load and above transportation purposes.

(10) $$TSR_{2}=\frac{{\bar{R}}_{T2}}{{\bar{R}}_{T1}}\times 100$$

where *TSR_2_* is the freeze–thaw splitting strength ratio (%). ${\bar{R}}_{T2}$ indicates the average value of splitting tensile strength of effective samples after freeze–thaw cycles (MPa). ${\bar{R}}_{T1}$ stands for the average value of splitting tensile strength of effective samples without freeze–thaw cycles (MPa).

### 2.3. Coring Sample Schemes

**Figure 3:**
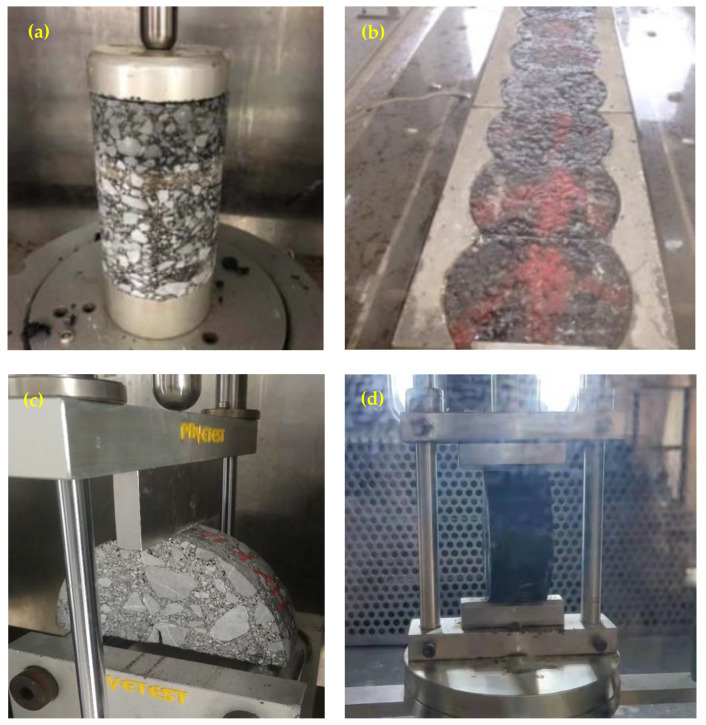
Appearance of core samples during different tests. (**a**) Dynamic creep test; (**b**) accelerated loading test; (**c**) semi-circular bending test; (**d**) splitting test.

The coring sample schemes for different tests are shown in Table 1. The core samples for the SCB test were cut into two semi-circles. The appearance of coring samples is shown in Figure 3.

## 3. Test Results and Analysis

### 3.1. Analysis of High-Temperature Stability

(1) Dynamic creep test

Table 2 presents the dynamic creep test results of core samples of different types of mixtures. In Table 2, for example 1#, the “#” stands for a symbol of specimen number, distinguishing that “1” is not a numerical value for test analysis (unless stated, the same below).

According to Table 2, the FN of overlay of SMA-13 stood at 439 times on average. Under a test temperature of 60 °C, the FN of cold recycling mixtures only reached 8, and the core samples of mixtures were damaged quickly. The FN increased to only 10~11, even when the temperature was changed to 55 °C. Therefore, the high-temperature stability of cold recycling mixtures was much lower than that of SMA overlay mixtures.

(2) MMLS3 loading test

**Figure 4:**
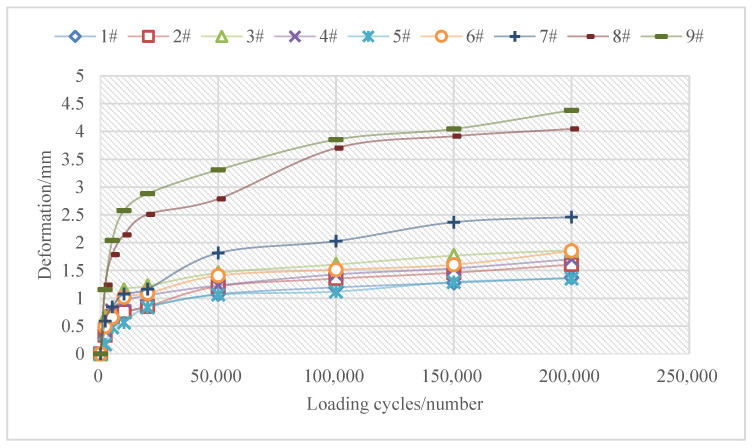
MMLS3 loading test results.

**Figure 5:**
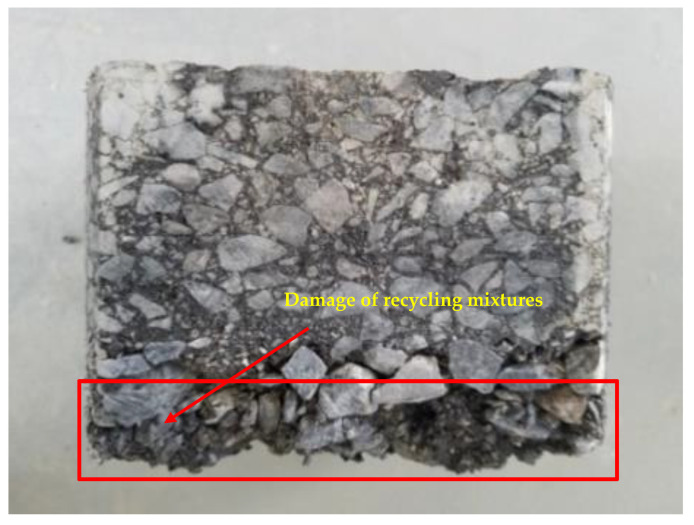
Appearance of cold recycling mixture after loading.

Figure 4 provides the MMLS3 accelerated loading test results of core samples of different types of mixtures, and Figure 5 presents the appearance of the core samples of cold recycling mixtures after loading. The deformation *RD*_10_ (after 100,000 times of loading) and the deformation rate *DS*_20−10_ (between 100,000 times and 200,000 times of loading) were calculated as shown in Table 3.

In Figure 4, although the deformation of both cold recycling mixtures and SMA-13 overlay mixtures was below 5 mm after 200,000 times of loading, this was not much large. The cold recycling mixtures started to drop particles under high temperature and hydrodynamic pressure, though complete loosening did not take place under the restraint of the mold. When the mold was removed at the end of the test, however, serious loosening occurred in the cold recycling mixtures (Figure 5). In addition, the high-temperature stability of SMA-13 overlay mixtures was better than that of cold recycling mixtures according to indicators *RD*_10_ and *DS*_20−10_, with the *RD*_10_ average value (1.5 mm) of SMA-13 overlay mixtures smaller than that (3.8 mm) of cold recycling mixtures and the *DS*_20−10_ average value (2.8 × 10^−3^ μm/cycle) of the former also smaller than that (4.4 × 10^−3^ μm/cycle) of the latter.

In summary, emulsified asphalt cold recycling mixtures were inferior to SMA-13 overlay mixtures in terms of high-temperature stability, which was attributed to the use of styrene-butadiene-styrene (SBS) modified asphalt and skeleton-dense gradation of the latter. Emulsified asphalt cold recycling mixtures were regenerated with the original upper layer (modified asphalt) and the middle layer (ordinary asphalt) and mixed with some modified emulsified asphalt and cement. Regardless of their asphalt performance or gradation composition, they were worse than the newly paved SMA mixtures. Furthermore, due to the slow moisture evaporation and cement hydration of emulsified asphalt, the strength of cold recycling mixtures formed in a longer time. Therefore, the poor strength was also a cause for their worse high-temperature performance compared to the SMA mixtures.

### 3.2. Analysis of Anti-Cracking Performance

**Figure 6:**
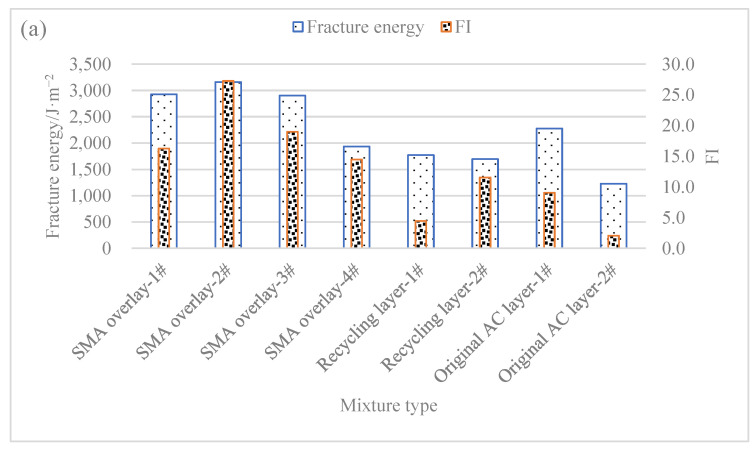

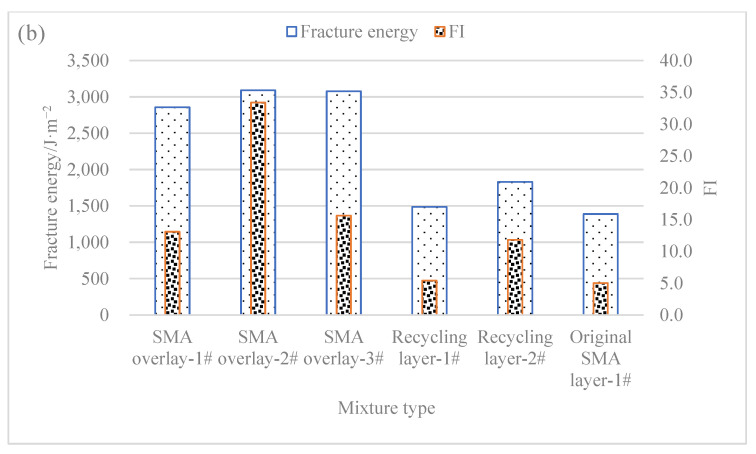
Semi-circular bending test results. (**a**) Fracture energy and FI of Section 1; (**b**) fracture energy and flexibility index of Section 2.

Figure 6 illustrates the SCB test results of core samples of different types of mixtures.

As shown in Figure 6, the best anti-cracking performance of the newly paved SMA mixtures was achieved with an average fracture energy of 2849 J/m^2^ and an average FI of 19.9. In contrast, the average fracture energy and FI of the cold recycling mixtures were 1696 J/m^2^ and 8.3, respectively, markedly lower than the newly paved SMA mixtures in fracture energy. Moreover, the average fracture energy and FI of the original AC layer mixtures was 1754 J/m^2^ and 5.6, respectively, while those of original SMA layer mixtures reached 1388 J/m^2^ and 5.0, respectively. Therefore, cold recycling mixtures were similar to original layer mixtures in anti-cracking performance.

As for the newly paved SMA mixtures, new SBS modified asphalt was adopted as the cementing material, featuring a high asphalt content and large filler-asphalt ratio, both of which can improve the anti-cracking performance. The serious aging condition of asphalt in emulsified asphalt cold recycling mixtures, together with the added cement, led to the decline in their anti-cracking performance.

### 3.3. Analysis of Moisture Susceptibility

#### 3.3.1. Effects of Curing Duration on Splitting Strength

**Figure 7:**
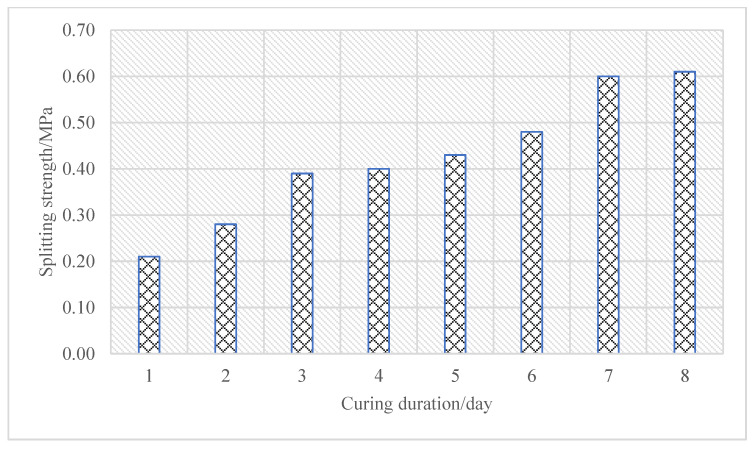
Splitting strength of cold recycling mixture under different curing duration.

Firstly, a set of core samples was drilled and taken every two days within eight days after the cold recycling layer of emulsified asphalt was formed, followed by the test of splitting strength, to analyze the effects of curing duration on the splitting strength of cold recycling mixtures. The splitting strength test results of the core samples of the cold recycling layer at 15 °C after different curing durations are given in Figure 7.

According to Figure 7, the splitting strength of cold recycling mixtures grew gradually as the duration of curing prolonged, suggesting that a proper curing duration was necessary for ensuring sufficient mechanical strength of cold recycling mixtures. The splitting strength development of cold recycling mixtures exhibited two phases depending on the formation law of splitting strength. The cold recycling mixtures welcomed the first stabilization phase on 3 d and the second one on 7 d of curing. A benchmark was set with the splitting strength of 0.60 MPa on 7 d of curing, thus the splitting strength on 1 d was 0.21 MPa, only 35% of the benchmark. The splitting strength stood at 0.39 MPa on 3 d, reaching 65% of the benchmark and achieving the early-stage strength to some degree.

#### 3.3.2. Moisture Susceptibility of Core Samples

**Figure 8:**
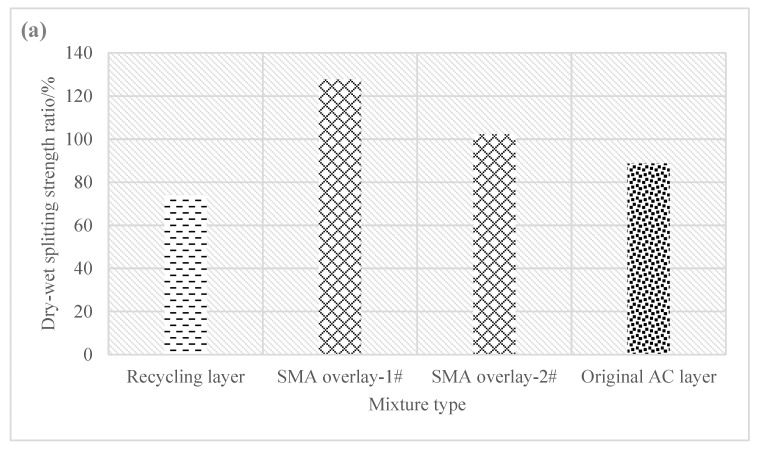

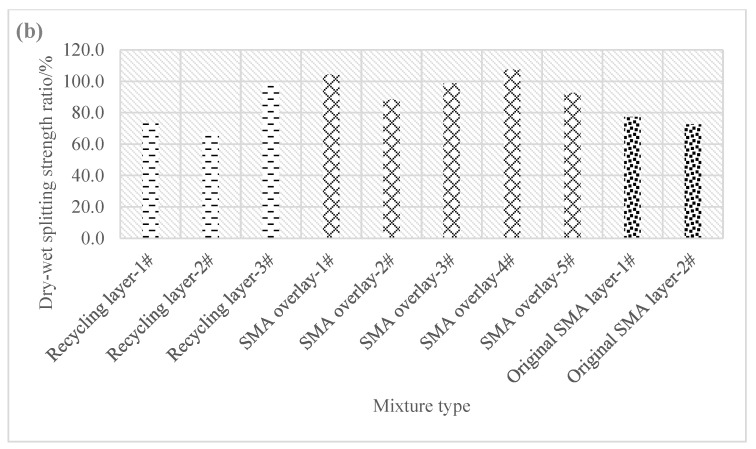
Dry–wet splitting test results. (**a**) Dry–wet splitting strength ratio of Section 1; (**b**) dry–wet splitting strength ratio of Section 2.

**Figure 9:**
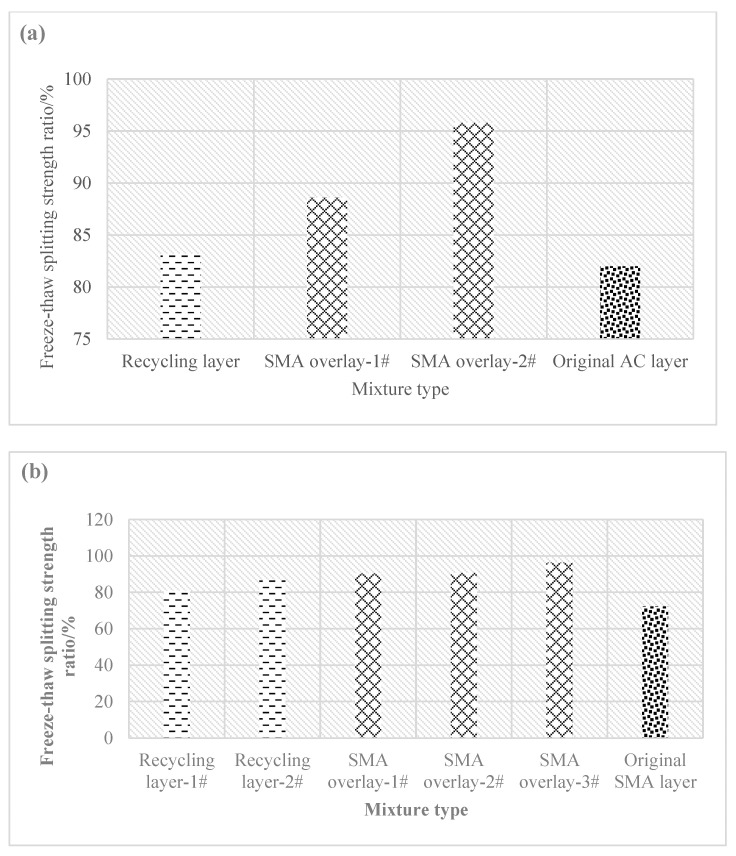
Freeze–thaw splitting test results. (**a**) Freeze–thaw splitting strength ratio of Section 1; (**b**) freeze–thaw splitting strength ratio of Section 2.

Figure 8 and Figure 9 show the dry–wet splitting strength ratio and the freeze–thaw splitting strength of different types of mixture of core samples.

As shown in Figure 8, the dry–wet splitting strength ratios of core samples of different types of mixtures varied greatly. In Pavement Section 1, the dry–wet splitting strength ratio was 74.1% for the cold recycling layer, 115% for the SMA overlay on average, and 88.7% for the original AC layer. In Pavement Section 2, the dry–wet splitting strength ratio was 80.1% for the cold recycling layer, 98.3% for the SMA overlay on average, and 75% for the original SMA layer. Therefore, the dry–wet splitting strength ratio of the emulsified asphalt cold recycling layer was significantly smaller than that of the SMA overlay but was close to that of the original pavement.

According to Figure 9, there was a wide difference among the freeze–thaw splitting strength ratios of the core samples of different types of mixtures as well. In Pavement Section 1, the freeze–thaw splitting strength ratio was 83.2% for the cold recycling layer, 92.2% for the SMA pavement layer on average, and 82% for the original AC layer. In Pavement Section 2, the freeze–thaw splitting strength ratio was 83.9% for the cold recycling layer, 92.4% for the SMA pavement layer on average, and 72.2% for the original SMA layer. As a result, the freeze–thaw splitting strength ratio of the emulsified asphalt cold recycling layer was also dramatically smaller than that of the SMA overlay and merely similar to that of the original pavement.

Based on the above analysis, the emulsified asphalt cold recycling mixture was much inferior to the newly paved SMA overlay but close to the original pavement in terms of moisture susceptibility, indicating that the cold in-place recycling technology cannot improve the moisture susceptibility of the original pavement. This was mainly attributed to the serious aging condition of the original asphalt in the cold recycling mixture, poor gradation and significant variability of the mixture, and unfavorable factors such as difficulties in the control of construction quality for large thickness recycling of the original pavement. Such adverse factors would lead to limited or failed effects of cold recycling technology on the moisture susceptibility of the original pavement. In the newly paved SMA overlay, the fresh SBS modified asphalt was used as the cementing material. In addition, the traits of a high asphalt content, a high filler–asphalt ratio, and the added fibers were all beneficial to the enhancement of moisture susceptibility.

According to the Specification of JTG/T 5521-2019 in China, in case of the use of emulsified asphalt cold recycling mixtures for heavy-load and above transportation purposes, the dry–wet splitting strength ratio shall be greater than 80% and the freeze–thaw splitting strength ratio shall exceed 75%. As shown by the test results of core samples drilled on site from the emulsified asphalt cold recycling pavement, the dry–wet splitting strength ratio of Pavement Section 1 did not meet the requirements, while that of Pavement Section 2 was merely qualified. However, the freeze–thaw splitting strength ratio of both sections satisfied the requirements. The wet–dry splitting strength ratio and the freeze–thaw splitting strength ratio, both of which are key control indicators for the quality of cold recycling mixtures, significantly affect the moisture susceptibility and anti-loosening performance of cold recycling pavements after they are put into service. The road project had been put into service for 6 months when the cores were taken, so the strength of the cold recycling mixtures had been further improved compared with the time when the pavement initially came into use. Nevertheless, the samples still failed the standards, indicating that the improvement of moisture susceptibility is a key issue for the use of cold recycling mixtures in high-layer recycling of asphalt pavement.

## 4. Conclusions

Two sections of emulsified asphalt cold in-place recycling pavement were selected in expressway practical engineering. By drilling core samples on the pavement, various pavement performances of the cold recycling mixture were analyzed through different test methods. The conclusions drawn are summarized as follows.

(1) The cold recycling layer was used as the middle layer of the maintenance pavement structure in high grade highway. Regardless of rutting resistance, cracking resistance, or moisture susceptibility, the comprehensive performances of cold recycling mixture were inferior to the newly paved SMA mixture.

(2) After opening to traffic 5 months, the cold recycling mixture of core samples loosened during the process of loading test, and the dry–wet splitting strength ratio failed the standards. The slow strength formation resulted in insufficient durability of the cold recycling mixture under high temperature and water immersion.

(3) The cement was used as a stabilizer in the cold recycling mixture. Adding a proper amount of stabilizer can improve the comprehensive performances of the cold recycling mixture. However, due to the performance attenuation and gradation deterioration of RAP materials, it is difficult for existing stabilizers to improve the performances to the level of fresh mixture. New types of stabilizers need to be developed to produce high performance cold recycling mixtures. Moreover, strength formation has a significant effect on the comprehensive performances of the cold recycling mixture. In the research, slow-setting emulsified asphalt and ordinary Portland cement were used. The effect of rapid-setting emulsified asphalt on the performances of cold recycling mixtures should be focused on, along with the early-strength type of cement. The size and voids of coring samples have significant influence on the test results. The volume parameters of coring samples should be considered to ensure more reliable test results.

## Figures and Tables

**Table 1:** Coring sample schemes of different tests.

Test Type	Core Sample Size	Section 1				Section 2			
		Traffic Lane		Emergence Lane		Traffic Lane		Emergence Lane	
Dynamic creep test	Diameter/cm	10		10		10		10	
	Height/cm	13		13		14		14	
	Component	Overlay + recycling layer		Overlay + original upper layer + original middle layer		Overlay + recycling layer		Overlay + original upper layer + original middle layer	
MMLS3 loading test	Diameter/cm	15		15		15		15	
	Height/cm	10		10		10		10	
	Component	Overlay + part of recycling layer		Overlay + original upper layer + part of original middle layer		Overlay + part of recycling layer		Overlay + original upper layer + part of original middle layer	
SCB test	Diameter/cm	15		15		15		15	
	Height/cm	4	5	4	4	4	5	4	4
	Component	Overlay	Part of recycling layer	Overlay	Original upper layer	Overlay	Part of recycling layer	Overlay	Original upper layer
Splitting test	Diameter/cm	10		10		10		10	
	Height/cm	4	5	4	4	4	5	4	4
	Component	Overlay	Part of recycling layer	Overlay	Original upper layer	Overlay	Part of recycling layer	Overlay	Original upper layer

**Table 2:** Dynamic creep test results of different core samples.

Section Type	Position	Mixture	Test Temperature/°C	Loading Cycles (Number)	FN (Number)
Section 1	Traffic lane	Recycling layer	60	42	8
	Emergence lane	Overlay of SMA-13	55	601	314
Section 2	Traffic lane	Recycling layer -1#	55	57	10
	Traffic lane	Recycling layer -2#	55	64	11
	Emergence lane	Overlay of SMA-13	55	900	564

**Table 3:** Calculation result of *RD*_10_, *DS*_20−10_.

Evaluation Index	Mixtures of SMA-13 Overlay							Cold Recycling Mixtures	
	Section 1				Section 2			Section 1	Section 2
	1#	2#	3#	4#	5#	6#	7#	8#	9#
*RD*_10_ (mm)	1.20	1.35	1.61	1.43	1.12	1.51	2.03	3.70	3.85
*DS*_20−10_ /(10^−3^ μm/cycle)	1.7	2.5	2.6	2.7	2.4	3.4	4.3	3.4	5.3

## Data Availability

Not applicable.

## References

[1] Xiao F., Yao S., Wang J., Li X., Amirkhanian S. (2018). A literature review on cold recycling technology of asphalt pavement. Constr. Build. Mater..

[2] Charmot S., Teh S.Y., Haris R.E.A., Ayob M.A., Ramzi M.R., Kamal D.D.M., Atan A. (2022). Field performance of bitumen emulsion Cold Central Plant Recycling (CCPR) mixture with same day and delayed overlay compared with traditional rehabilitation procedures. Case Stud. Constr. Mater..

[3] Marinković M., Zavadskas E.K., Matić B., Jovanović S., Das D.K., Sremac S. (2022). Application of wasted and recycled materials for production of stabilized layers of road structures. Buildings.

[4] Vaitkus A., Gražulytė J., Baltrušaitis A., Židanavičiūtė J., Čygas D. (2021). Long-term performance of pavement structures with cold in-place recycled base course. Balt. J. Road Bridge Eng..

[5] Liu Z., Sun L., Zhai J., Huang W. (2022). A review of design methods for cold in-place recycling asphalt mixtures: Design processes, key parameters, and evaluation. J. Cleaner Prod..

[6] Saidi A., Ali A., Lein W., Mehta Y. (2019). A balanced mix design method for selecting the optimum binder content of cold in-place recycling asphalt mixtures. Transp. Res. Rec..

[7] Ayala F.C., Sebaaly P.E., Hand A.J., Hajj E.Y., Baumgardner G. (2021). Performance characteristics of cold in-place recycling mixtures. J. Mater. Civ. Eng..

[8] Schroeder R.L. (1994). The use of recycled materials in highway construction. Public Roads.

[9] Kuchiishi A.K., Vasconcelos K., Bariani Bernucci L.L. (2021). Effect of mixture composition on the mechanical behaviour of cold recycled asphalt mixtures. Int. J. Pavement Eng..

[10] Euch Khay S.E., Euch Ben Said S.E., Loulizi A., Neji J. (2015). Laboratory investigation of cement-treated reclaimed asphalt pavement material. J. Mater. Civ. Eng..

[11] Ma B., Wang H., Wei D. (2011). Performance of RAP in the system of cold inplace recycling of asphalt pavement. J. Wuhan Univ. Technol. Mater. Sci. Ed..

[12] Kim Y., Lee H.D. (2012). Performance evaluation of Cold In-Place Recycling mixtures using emulsified asphalt based on dynamic modulus, flow number, flow time, and raveling loss. KSCE J. Civ. Eng..

[13] Ameri M., Behnood A. (2012). Laboratory studies to investigate the properties of CIR mixes containing steel slag as a substitute for virgin aggregates. Constr. Build. Mater..

[14] Andavan S., Kumar B.M. (2020). Case study on soil stabilization by using bitumen emulsions—A review. Mater. Today Proc..

[15] Yaghoubi E., Ghorbani B., Saberian M., van Staden R., Guerrieri M., Fragomeni S. (2023). Permanent deformation response of demolition wastes stabilised with bitumen emulsion as pavement base/subbase. Transp. Geotech..

[16] Gao L., Ni F., Braham A., Luo H. (2014). Mixed-mode cracking behavior of cold recycled mixes with emulsion using arcan configuration. Constr. Build. Mater..

[17] Wang H.F., Ma B.G., Yin X.B. (2010). Mechanical property effect of Na_2_SO_4_ on cement-reclaimed asphalt pavement mixture. Adv. Mater. Res..

[18] Gao L., Ni F., Charmot S., Li Q. (2014). High-temperature performance of multilayer pavement with cold in-place recycling mixtures. Road Mater. Pavement Des..

[19] Ogbo C., Dave E., Sias J. (2022). Laboratory investigation of factors affecting the evolution of curing in cold in-place recycled materials. Transp. Res. Rec..

[20] Graziani A., Grilli A., Mignini C., Balzi A. (2022). Assessing the field curing behavior of cold recycled asphalt mixtures. Adv. Mater. Sci. Eng..

[21] Pérez I., Gómez-Meijide B., Pasandín A.R., García A., Airey G. (2021). Enhancement of curing properties of cold in-place recycling asphalt mixtures by induction heating. Int. J. Pavement Eng..

[22] Saidi A., Ali A., Mehta Y., Decarlo C.J., Elshaer M. (2022). Field assessment of cold in-place recycled asphalt mixtures using accelerated pavement testing. J. Transp. Eng. Part B Pavements.

[23] Orosa P., Perez I., Pasandin A.R. (2022). Evaluation of the shear and permanent deformation properties of cold in-place recycled mixtures with bitumen emulsion using triaxial tests. Constr. Build. Mater..

[24] Teshale E.Z., Rettner D., Hartleib A., Kriesel D. (2017). Application of laboratory asphalt cracking tests to cold in-place recycled mixtures. Road Mater. Pavement Des..

[25] Kaseer F., Yin F., Arámbula-Mercado E., Martin A.E., Daniel J.S., Salari S. (2018). Development of an index to evaluate the cracking potential of asphalt mixtures using the semi-circular bending test. Constr. Build. Mater..

[26] Safazadeh F., Romero P., Mohammad Asib A.S., VanFrank K. (2022). Methods to evaluate intermediate temperature properties of asphalt mixtures by the semi-circular bending (SCB) test. Road Mater. Pavement Des..

[27] Saha G., Biligiri K.P. (2019). Novel procedural pragmatics of dynamic Semi-Circular Bending test for fatigue evaluation of asphalt mixtures. Road Mater. Pavement Des..

[28] Sabouri M., Wegman D.E. (2022). Performance evaluation of cold in-place recycling materials through a simple semi-circular bending test. Road Mater. Pavement Des..

[29] Dolzycki B., Szydlowski C., Jaczewski M. (2020). The influence of combination of binding agents on fatigue properties of deep cold in-place recycled mixtures in Indirect Tensile Fatigue Test (ITFT). Constr. Build. Mater..

[30] Zhao H., Ren J., Chen Z., Luan H., Yi J. (2021). Freeze and thaw field investigation of foamed asphalt cold recycling mixture in cold region. Case Stud. Constr. Mater..

[31] Lyu Z., Shen A., Qin X., Yang X., Li Y. (2019). Grey target optimization and the mechanism of cold recycled asphalt mixture with comprehensive performance. Constr. Build. Mater..

[32] Cheng P., Yi J., Chen Z., Luan H., Feng D. (2022). Influence factors of strength and performance of foamed asphalt cold recycled mixture. Road Mater. Pavement Des..

[33] Wang D., Guo T., Chang H., Yao X., Chen Y., Wang T. (2021). Research on the performance of regenerant modified cold recycled mixture with asphalt emulsions. Sustainability.

[34] Du S. (2021). Effect of different fibres on the performance properties of cold recycled mixture with asphalt emulsion. Int. J. Pavement Eng..

[35] (2017). Standard Method of Test for Determining the Dynamic MODULUS and Flow number for Asphalt Mixtures Using the Asphalt Mixture Performance Tester (AMPT).

[36] Bhattacharjee S., Gould J., Mallick R.B., Hugo F. (2004). An evaluation of use of accelerated loading equipment for determination of fatigue response of asphalt pavement in laboratory. Int. J. Pavement Eng..

[37] (2016). Standard Method of Test For Determining the Fracture Potential of Asphalt Mixtures Using Semicircular Bend Geometry (Scb) At Intermediate Temperature.

[38] (2011). Standard Test Methods of Bitumen and Bituminous Mixtures for Highway Engineering.

